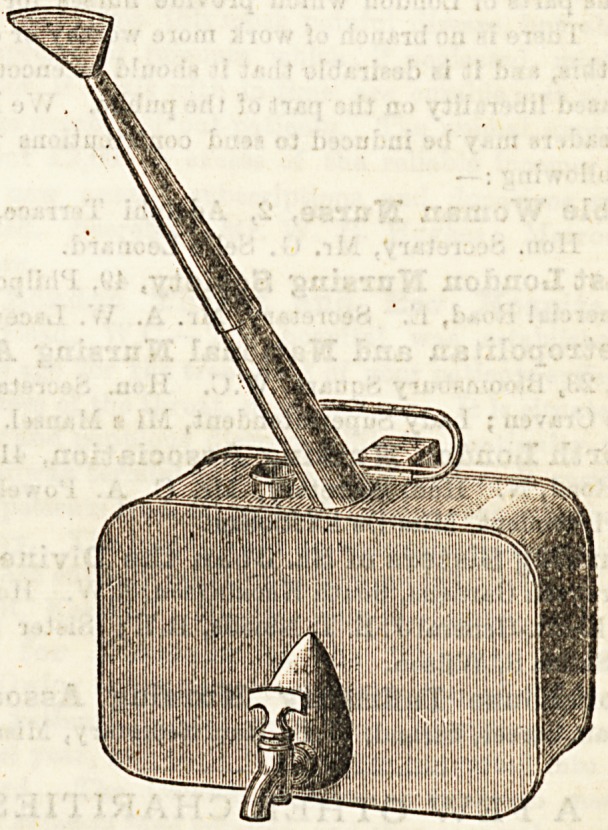# New Drugs, Appliances, and Things Medical

**Published:** 1891-12-19

**Authors:** 


### NEW DRUGS, APPLIANCES, AND THINGS MEDICAL.

[All preparations' appliances, novelties, etc., of which a notice is desired, should be sent for The Editor, to care of The Manager, 140, Strand, London, W.C.]

### AN EXCELLENT BRONCHITIS KETTLE.

The bronchitis kettle is now considered such an essential in every household, that makers are directing especial attention to their perfection. The one before us, supplied by Messrs. Benham and Froud, Chandos Street, Strand, is of excellent structure. It will contain sufficient water to render frequent re-filling unnecessary, and the spout aperture is of such a form as to deliver a large volume of steam into the room. The main feature, however, is the hook attached to the kettle, by means of which it can be fixed in front of the fire, so that the fire can be replenished without removing the kettle, and a tap is provided which minimises the danger of upsetting, and permits hot water to be withdrawn. There are three shapes—round, oval, and oblong. The prices range for these from 2s. 9d. to 6s. 9d.

### SALVINE DENTIFRICE.

Salvine, a dentifrice prepared by Dr. H. Wren Oliver, and sold at 3, Oxford Street, can be confidently recommended to our readers. The preparation possesses anti-acid, antiseptic, and astringent qualities. It claims to assist in the preservation of the teeth from decay, and in use is very agreeable and refreshing. It is sold in tubes at 1s., 1s. 6d., and 2s. 6d.

## Figures and Tables

**Figure f1:**